# Human umbilical cord-derived mesenchymal stem cells prevent the progression of early diabetic nephropathy through inhibiting inflammation and fibrosis

**DOI:** 10.1186/s13287-020-01852-y

**Published:** 2020-08-03

**Authors:** E Xiang, Bing Han, Quan Zhang, Wei Rao, Zhangfan Wang, Cheng Chang, Yaqi Zhang, Chengshu Tu, Changyong Li, Dongcheng Wu

**Affiliations:** 1 Department of Biochemistry and Molecular Biology, Wuhan University School of Basic Medical Sciences, Wuhan, China; 2 Wuhan Hamilton Biotechnology Co., Ltd, Wuhan, China; 3 Department of Physiology, Wuhan University School of Basic Medical Sciences, Wuhan, China

**Keywords:** Diabetic nephropathy, Umbilical cord mesenchymal stem cells, Inflammation, Renal fibrosis

## Abstract

**Background:**

Diabetic nephropathy (DN) is one of the most serious complications of diabetes and the leading cause of end-stage chronic kidney disease. Currently, there are no effective drugs for treating DN. Therefore, novel and effective strategies to ameliorate DN at the early stage should be identified. This study aimed to explore the effectiveness and underlying mechanisms of human umbilical cord mesenchymal stem cells (UC-MSCs) in DN.

**Methods:**

We identified the basic biological properties and examined the multilineage differentiation potential of UC-MSCs. Streptozotocin (STZ)-induced DN rats were infused with 2 × 10^6^ UC-MSCs via the tail vein at week 6. After 2 weeks, we measured blood glucose level, levels of renal function parameters in the blood and urine, and cytokine levels in the kidney and blood, and analyzed renal pathological changes after UC-MSC treatment. We also determined the colonization of UC-MSCs in the kidney with or without STZ injection. Moreover, in vitro experiments were performed to analyze cytokine levels of renal tubular epithelial cell lines (NRK-52E, HK2) and human renal glomerular endothelial cell line (hrGECs).

**Results:**

UC-MSCs significantly ameliorated functional parameters, such as 24-h urinary protein, creatinine clearance rate, serum creatinine, urea nitrogen, and renal hypertrophy index. Pathological changes in the kidney were manifested by significant reductions in renal vacuole degeneration, inflammatory cell infiltration, and renal interstitial fibrosis after UC-MSC treatment. We observed that the number of UC-MSCs recruited to the injured kidneys was increased compared with the controls. UC-MSCs apparently reduced the levels of pro-inflammatory cytokines (IL-6, IL-1β, and TNF-α) and pro-fibrotic factor (TGF-β) in the kidney and blood of DN rats. In vitro experiments showed that UC-MSC conditioned medium and UC-MSC-derived exosomes decreased the production of these cytokines in high glucose-injured renal tubular epithelial cells, and renal glomerular endothelial cells. Moreover, UC-MSCs secreted large amounts of growth factors including epidermal growth factor, fibroblast growth factor, hepatocyte growth factor, and vascular endothelial growth factor.

**Conclusion:**

UC-MSCs can effectively improve the renal function, inhibit inflammation and fibrosis, and prevent its progression in a model of diabetes-induced chronic renal injury, indicating that UC-MSCs could be a promising treatment strategy for DN.

## Background

Diabetic nephropathy (DN) is the most detrimental microvascular complication of diabetes and the leading cause of chronic kidney disease worldwide. Diabetes is a progressive disease. Long-term hyperglycemia causes damage to tissues and organs, resulting in various diabetic complications, such as diabetic retinopathy, diabetic foot, DN, and so on [1]. Among them, DN is a refractory disease with low awareness, high incidence, and high disability. The incidence of DN can reach 30 to 40% after 20 years of diabetes, of which 5~10% of patients will progress to end-stage renal disease, and epidemiological surveys predict that by 2030, DN will become the seventh leading cause of death in the world [2–5]. Risk factors for DN include advanced age, gender, long disease course, obesity, high salt diet, dyslipidemia, nephrotoxic substances, acute kidney injury, and excessive protein intake. Hyperglycemia and hypertension are the most significant risk factors [6, 7]. Compared with other types of diabetic patients, DN patients have a higher mortality rate, and most of the deaths are due to cardiovascular events [8].

The main manifestation of early DN is the appearance of microalbuminuria. As the disease progresses, a large amount of proteinuria appears in most patients with DN and eventually develops into chronic renal failure until uremia [9]. The main pathological features of DN include glomerular basement membrane thickening, mesangial expansion, and glomerular sclerosis. Besides, podocyte loss and apoptosis, interstitial inflammation infiltration, renal interstitial fibrosis, and renal tubular atrophy sparse capillaries around the tube are also included in the pathological characteristics of DN [10]. The prevention and treatment of DN are mainly divided into pre-diabetes prevention (active screening, early detection, and reasonable intervention), early treatment (tight control of blood glucose and blood pressure) to delay the development of DN and comprehensive treatment of advanced DN (including alternative treatments such as dialysis or kidney transplantation) to reduce the risk of cardiovascular events and death [11]. Currently, effective therapeutic strategies to counteract and reverse the progression of DN are lacking; therefore, it is imperative to develop new strategies for treating DN.

Notably, stem cell therapy has become the most likely new breakthrough in the treatment of DN due to its self-renewal capacity, multilineage differentiation potential, paracrine effects, and immunomodulatory properties. Mesenchymal stem cells (MSCs) are a class of adult stem cells derived from mesoderm that have strong self-renewal and multi-directional differentiation potential. The main sources are the bone marrow MSCs (BM-MSCs), adipose-derived MSCs (AD-MSCs), and umbilical cord MSCs (UC-MSCs). A large number of studies have investigated the role of MSCs in many diseases, including diabetic retinopathy [12], myocardial infarction [13], diabetes [14], and DN [15–19] in mice and rats. However, the underlying mechanisms of these beneficial effects are not completely elucidated.

UC-MSCs are considered to be a better choice of MSCs for clinical applications due to their easy collection, low immunogenicity, and high paracrine potential [20]. In this study, we established streptozotocin (STZ)-induced DN rat models and high glucose-induced HK2 cells (human proximal tubular epithelial cells), NRK-52E cells (rat renal tubular epithelial cells), and hrGECs (human renal glomerular endothelial cells) injury models in vitro and explored the therapeutic effect and underlying mechanisms of UC-MSCs in DN. Our results suggested that early transplantation of UC-MSCs holds a promising role in controlling the progress of DN at an early stage. This study could provide preclinical experimental data for the development of new stem cell drugs for the treatment of DN.

## Materials and methods

### Isolation, culture, and identification of UC-MSCs

The use of the human umbilical cord tissue from a healthy donor who gave birth and signed informed consent in Renmin Hospital was supported by the Institutional Ethics Review Board of Renmin Hospital of Wuhan University (Permit Number: WDRY2019-G001). In a sterile Petri dish, the umbilical cord was cut into small pieces of about 1.0 mm. The tissue fragments were washed with PBS and centrifuged at 1900 r/min for 6 min. The tissue fragments were resuspended with 25 mL of serum-free stem cell culture medium (Lonza, MD, Walkersville), inoculated into a T175 culture flask, and placed in an incubator at 37 °C and 5% CO_2_ concentration. The primary cells were harvested when the confluence reached 80%. When the adherent cells were passaged to the fourth generation, after 72 h of cell culture, the supernatant was collected as UC-MSC-conditioned medium (UC-MSC-CM) and centrifuged at 500 g for 10 min and passed through a 0.22-μm filter (Millipore) before use. The fifth generations of cells were used in this animal experiment. Human UC-MSC induction differentiation experiments were carried out using Oil red O staining to confirm adipogenesis, using Alizarin red staining to verify osteogenesis, and Alcian blue staining to examine chondrogenesis. Related cell surface markers, such as CD19, CD34, CD45, HLA-DR, CD105, CD90, CD44, and CD73 (Biolegend, USA), were detected by flow cytometry.

### Rat DN model and treatment

Specific pathogen-free male Sprague-Dawley rats (180–200 g; 6 weeks old) from Hubei provincial center for disease control and prevention (Wuhan, China) were used in this research. This study was performed in accordance with Guidelines for the Care and Use of Laboratory Animals and the Animal Welfare Act in China and approved by the Committee of Animal Care and Use of Hubei Provincial Center for Food and Drug Safety Evaluation and Animal Experiment (Permit Number: 20200101). Rats were maintained under standard feeding conditions (temperature 20 ± 2 °C; humidity 45–55%; 12 h light & dark cycle) and free access to food and water.

All rats were randomly allocated to the control group (*N* = 5) or diabetic group (*N* = 10). We used a well-established rat model of DN induced by STZ, as described [21]. Briefly, the diabetic rats were induced by a single intraperitoneal injection of 60 mg/kg STZ (Sigma-Aldrich, MO, St. Louis) (dissolved in 0.1 M citrate buffer, pH 4.5). The control group received an equal volume of vehicle treatment. The tail blood glucose concentration of rat ≥ 16.7 mmol/L for 3 consecutive days was confirmed as diabetes. The rat urine was collected using metabolic cages to measure volume and protein concentration on weeks 4 to 6 after STZ treatment. The 24-h urinary protein ≥ 30 mg/24 h was verified as DN. Then, all DN rats were randomly divided into DN + PBS group (*N* = 5) and DN + UC-MSC group (*N* = 5). In DN + UC-MSC group, 2 × 10^6^/500 μL UC-MSCs were injected via tail vein, while the DN + PBS group and control group were received the same volume PBS. Two weeks after UC-MSC treatment, urine samples were collected, and all rats were anesthetized with 2 to 3% isoflurane and euthanized. Urine samples were used to detect 24 h-urinary protein and urine creatinine (Ucr), and the blood samples were used to measure serum creatinine (Scr) and blood urea nitrogen by assay kits. The creatinine clearance rate (Ccr) was then calculated (Ccr = Ucr/Scr × V, V: ml/min, urine volume per minute). Kidney tissues were collected for further analysis. Rat DN model and treatment were performed by a single experienced operator in a blinded manner. Completely randomized design and blinding were adopted in animal experiments.

### Histological analysis, immunofluorescence, and immunohistochemistry staining

The kidney was cut longitudinally along the long axis, fixed with 10% neutral formalin, embedded in paraffin, and cut into 5-μm-thick slices several times in a row. To detect the degree of pathological damage and fibrosis of kidney tissues, hematoxylin and eosin (H&E), periodic acid-Schiff (PAS), and Masson’s trichrome (Masson) staining were used to observe the morphological changes of the kidneys under a light microscope.

For immunofluorescence staining, kidney paraffin slices were dewaxed into water. Then, the antigen was retrieved with EDTA antigen retrieval buffer (pH 8.0). Next, slices were washed with PBS (pH 7.4). After slightly drying, the autofluorescence quencher was added to slices, and the slices were rinsed. BSA was dropwise added to slices and incubated. Then, slices were incubated at 4 °C overnight with 1:100 diluted rabbit anti-F4/80 antibody, 1:100 diluted rabbit anti-Collagen IV antibody and 1:100 diluted rabbit anti-CD3 (Bioss, China), respectively. After washing with PBS, the corresponding secondary antibody (Boster, China) was added to incubate at room temperature for 50 min in dark. After DAPI staining performing (Solarbio, China), images were detected by a fluorescence microscope (Eclipse Ci-e, Nikon, Japan).

For immunohistochemistry staining, kidney sections were blocked with 3% H_2_O_2_ in absolute methanol for 30 min and nonspecific sites were blocked with bovine serum albumin (BSA) for 30 min at room temperature. Then, slices were incubated overnight at 4 °C with 1:200 diluted mouse anti-α-SMA antibody (Boster, China), 1:100 diluted rabbit TGF-β antibody (Bioss, China), and 1:50 diluted MAB1281 antibody (EMD Millipore, USA), respectively. After that, the related goat anti-mouse/rabbit IgG secondary antibody (HRP-labeled) (Boster, China) was added dropwise and incubated. After washing with PBS and counterstaining with 3, 3′-diamnobenzidine (DAB), then counterstaining with cell nuclei hematoxylin, slices were placed in xylene, concentration gradient alcohol to dehydrate, and images were examined by a light microscope.

### Enzyme-linked immunosorbent assay

The levels of IL-1β, IL-6, TGF-β, and TNF-α in kidney tissues, serum, HK2 cells, NRK-52E cells, and hrGECs were determined. The levels of epidermal growth factor (EGF), fibroblast growth factor (FGF), hepatocyte growth factor (HGF), and vascular endothelial growth factor (VEGF) were detected in UC-MSC-CM and UC-MSC-Exo. ELISA kits (Mlbio, Shanghai, China) were used following the manufacturer’s instructions.

### RNA extraction and RT-qPCR assay

Total RNA was extracted from the kidneys, HK2 cells, NRK-52E cells, and hrGECs using Trizol reagent (Life Technologies, USA). The methods of reverse transcription and RT-qPCR were performed following the manufacturer’s instructions (RNA-Solv Reagent). The primer sequences for RT-qPCR are listed in Additional file 1: Table 1 and Table 2. The experimental data were normalized to endogenous housekeeping gene GAPDH with 2^−ΔΔCt^ method.

### Western blot analysis

About 100 mg of the kidney tissue was lysed in 1 mL ice-cold RIPA lysis buffer (Beyotime, China) with 1 mM PMSF. The concentration of protein was examined by BCA assay kits. About 50 μg protein samples were loaded in 10% SDS-PAGE gels and transferred to PVDF membrane (Millipore, USA). After blocking with 5% skim milk for 1 h, the membrane was respectively incubated overnight at 4 °C with TGF-β (1:1000, Abcam, USA), GAPDH (1:5000, Bioss, China), CD9 (1:1000, Proteintech, USA), Alix (1:1000, Proteintech, USA), and Calnexin (1:1000, Cell Signaling, USA), subsequently washed and incubated with corresponding secondary antibodies for 1 h at room temperature. Specific protein bands were visualized applying ECL kit.

### Isolation and identification of UC-MSC-Exo

Exosomes isolation and identification were performed as described [22] with modifications. The more detailed description of the exosomes isolation is available in Additional Fig. 2. Exosomes extracted from UC-MSCs (UC-MSC-Exo) were observed by transmission electron microscope (TEM) (Tecnai G2 Spirit Bio TWIN, FEI, USA). Particle size and concentrations were measured by nanoparticle tracking analysis (NTA) (Particle Metrix, Germany). Related markers (CD9, Alix) and negative control (Calnexin) of exosomes were detected by western blot.

### Cell culture and treatment

HK2 cells (human proximal tubular epithelial cells), NRK-52E cells (rat renal tubular epithelial cells), and hrGECs (human renal glomerular endothelial cells) were purchased from the China Center for Type Culture Collection (Wuhan China). HK2 cells were cultured in RPMI 1640 medium containing 10% fetal bovine serum (FBS; Hyclone, USA), NRK-52E cells were cultured in DMEM-low glucose medium with 10% FBS, and hrGECs were cultured in DMEM/F12 containing 10% FBS at 37 °C and 5% CO_2_ concentration in a humidified atmosphere. Cells were seeded into 6-well plates and randomly divided into eight groups: (1) low glucose (LG) group: d-glucose 5.5 mmol/L; (2) high glucose (HG) group: d-glucose 30 mmol/L; (3) HG + 25% UC-MSC-CM group; (4) HG + 50% UC-MSC-CM group; (5) HG + 100% UC-MSC-CM group; (6) HG + 25 μg/mL UC-MSC-Exo group; (7) HG + 50 μg/mL UC-MSC-Exo group; and (8) HG + 100 μg/mL UC-MSC-Exo group. After 24 h of co-culture, the supernatant and cells were collected for further analysis.

### Statistical analysis

All data are presented as mean ± S.E.M by Graph Pad prism 5 and were analyzed by unpaired Student’s *t* test with SPSS 19 statistical software (SPSS Inc., Chicago, Illinois). Multiple group comparisons were made using one-way analysis of variance (ANOVA) followed by Bonferroni’s post hoc test. *P* value < 0.05 was considered significant.

## Results

### Induced differentiation ability and biological properties of UC-MSCs

**Fig. 1:**
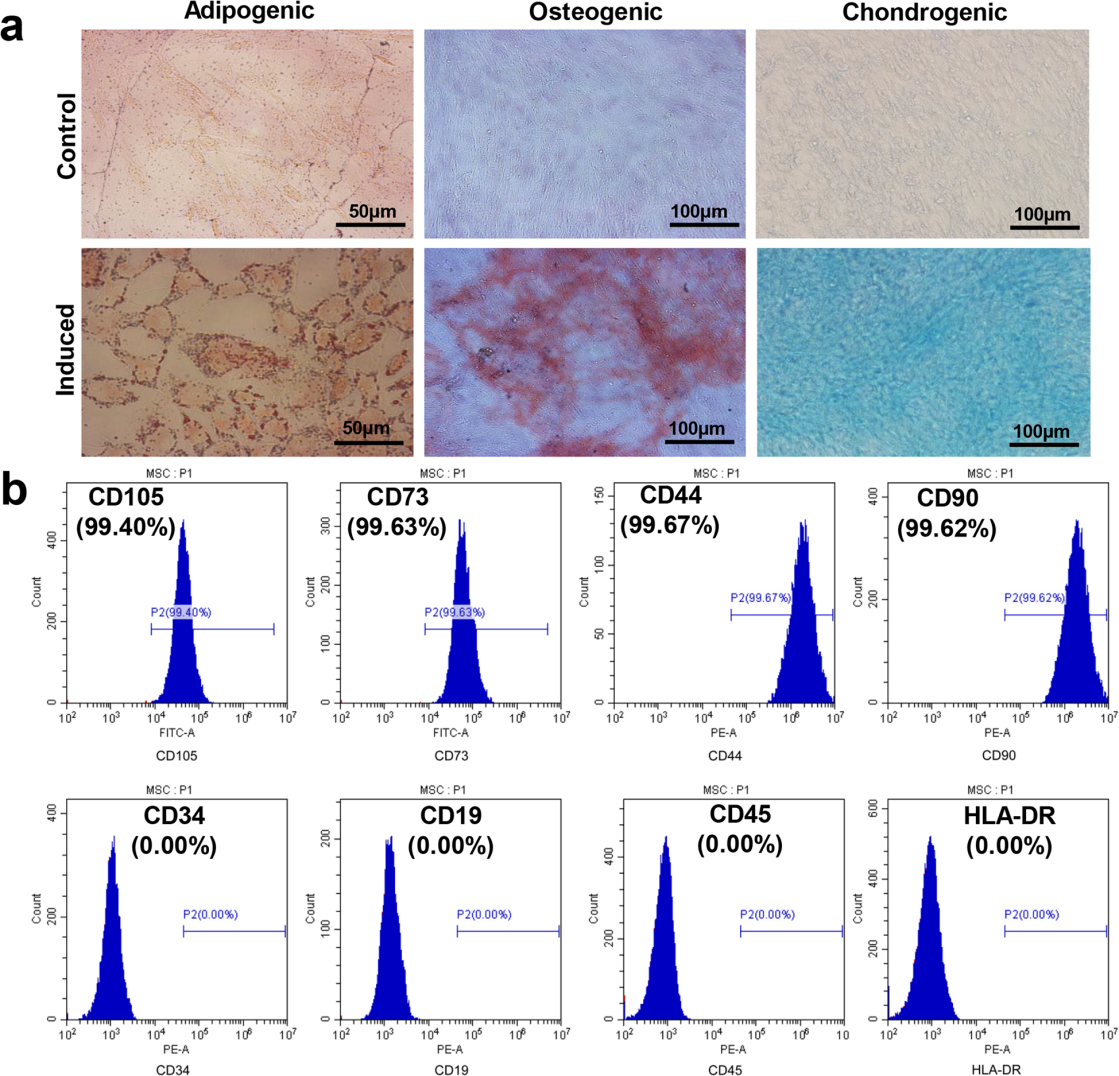
Induced differentiation ability and characteristic surface markers of UC-MSCs. **a** Differentiation abilities of cells were detected by cellular staining. The order from left to right: adipogenesis using Oil red O staining, osteogenesis using Alizarin red staining, and chondrogenesis using Alcian blue staining. **b** Specific surface markers of cells were examined by flow cytometry. The UC-MSCs associated with markers were positive for CD105, CD90, CD44, and CD73 and were negative for CD19, CD34, CD45, and HLA-DR

UC-MSCs were derived from umbilical cord tissues, which had various inducing differentiation capabilities and basic cell biological properties. Biological effectiveness experiments confirmed that UC-MSCs could be differentiated into adipogenic, osteogenic, and chondrogenic phenotypes (Fig. 1a). Flow cytometry experiments confirmed that UC-MSCs were positive for CD105 (99.40%), CD90 (99.63%), CD44 (99.67%), CD73 (99.62%), and negative for CD19 (0.00%), CD34 (0.00%), CD45 (0.00%), and HLA-DR (0.00%) (Fig. 1b).

### In vivo model of DN was induced by STZ

**Fig. 2:**
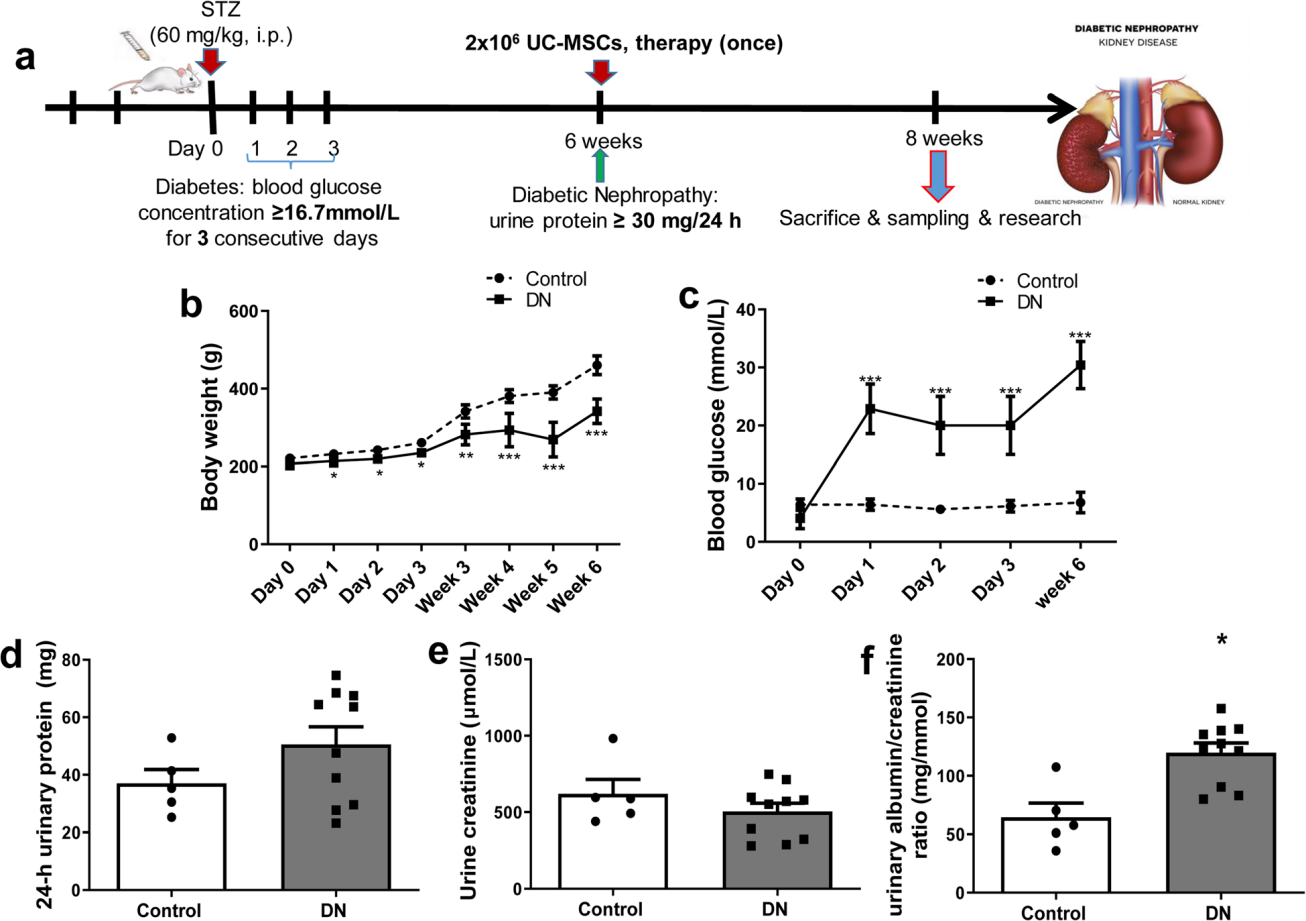
Timetable and flowchart of rat treatment and cell therapy as well as the identification of rat DN model. **a** The flowchart of rat treatment from day 0 to week 8. **b** The growth curve of body weight. **c** Blood glucose concentration curve. **d** 24-h urinary protein. **e** Urine creatinine. **f** Urinary albumin/creatinine ratio. *N* = 5 (control), *N* = 10 (DN), data are presented as mean ± SEM. *P < 0.05, **P < 0.01, ***P < 0.001 vs control group

To explore the therapeutic effect of UC-MSCs on DN, we established a rat model of DN induced by STZ injection. Animals were sacrificed after 2 weeks of treatment, and specimens were collected for further analysis (Fig. 2a). After STZ processing, the bodyweight growth of DN group was significantly lower than that of control group from day 1 to week 6 (Fig. 2b). Besides, the blood glucose levels of DN group were greater than 16.7 mmol/L and were significantly higher than that of the control rats (Fig. 2c). The 24-h urinary protein of DN rats was increased and exceeded 30 mg/24 h at week 6 (Fig. 2d), but the Ucr was decreased (Fig. 2e). Meanwhile, the urinary albumin/creatinine ratio of DN group was significantly increased (Fig. 2f).

### UC-MSC treatment improved renal function of DN rats

**Fig. 3:**
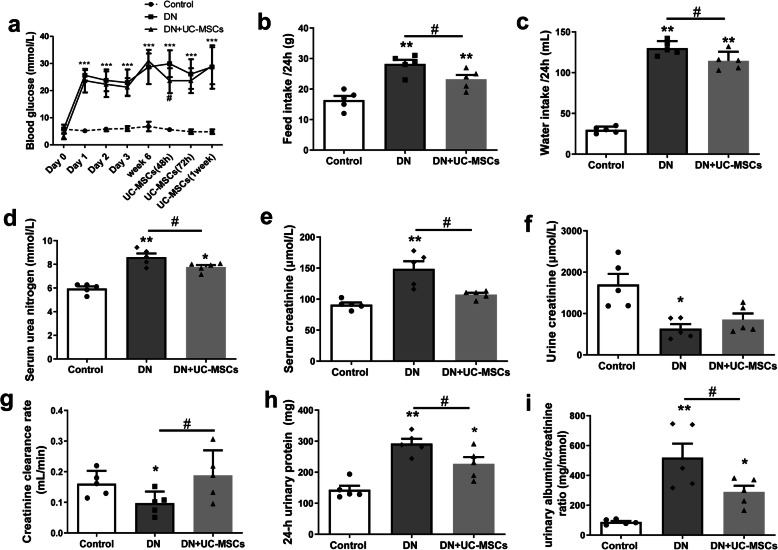
Effects of UC-MSC treatment on biochemical indexes of DN rats. **a** Blood glucose concentration curve. **b** Feed intake/24 h**. c** Water inkake/24 h. **d** Serum urea nitrogen. **e** Serum creatinine. **f** Urine creatinine. **g** Creatinine clearance rate. **h** 24-h urinary protein. **i** Urinary albumin/creatinine ratio. *N* = 5 (per group), data are presented as mean ± SEM. *P < 0.05, **P < 0.01, ***P < 0.001 vs control. ^#^*P* < 0.05 vs DN group

In the process of DN modeling and UC-MSC intervention, we observed that DN rats had significantly increased feed and water intake, compared to the control group. Compared with DN rats, UC-MSC-treated DN rats showed a reduction in feed and water intake (Fig. 3b, c). After UC-MSC transplantation, no significant decrease was detected in the blood glucose concentration in DN rats (Fig. 3a). The serum urea nitrogen and Scr of DN group were greatly increased than those of the control group, while UC-MSC treatment significantly suppressed the increase (Fig. 3d, e). Compared with DN group, UC-MSC treatment obviously suppressed the increase in 24-h urinary protein (Fig. 3h) and urinary albumin/creatinine ratio (Fig. 3i), and evidently upregulated Ccr (Fig. 3g). There was a modest but insignificant increase in urine creatinine level on week 8 after UC-MSC infusion (Fig. 3f). These results indicated that UC-MSCs effectively improved renal function in DN rats.

### UC-MSC treatment ameliorated renal pathological changes

**Fig. 4:**
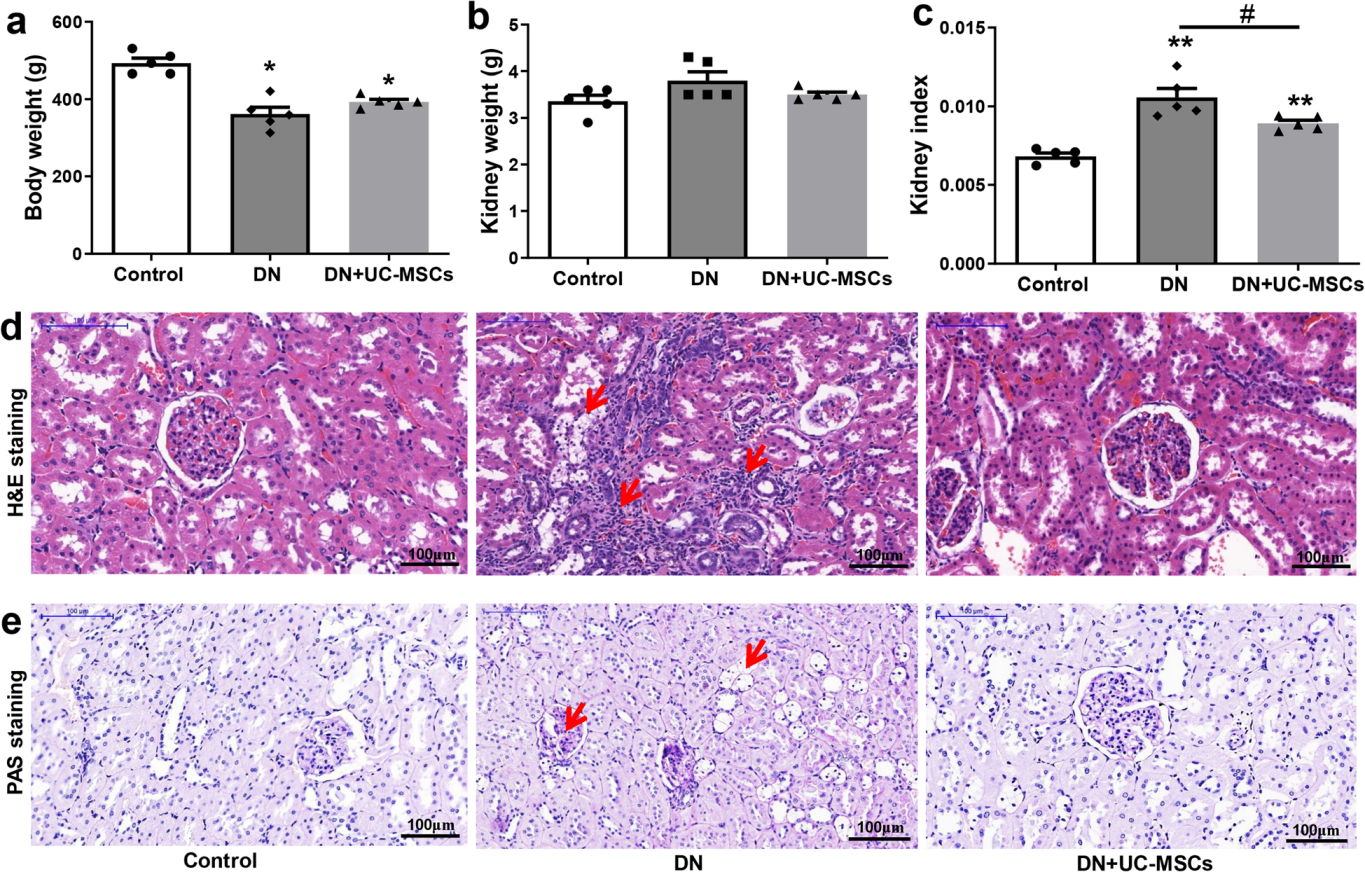
Histopathology analysis of tissues from DN rats. **a** Bodyweight. **b** Kidney weight. **c** Kidney index (kidney weight/bodyweight). **d** Hematoxylin-eosin (H&E) staining. **e** Periodic acid-Schiff (PAS) staining. *N* = 5 (per group), data are presented as mean ± SEM. *P < 0.05, **P < 0.01 vs control. ^#^*P* < 0.05 vs DN group

**Fig. 5:**
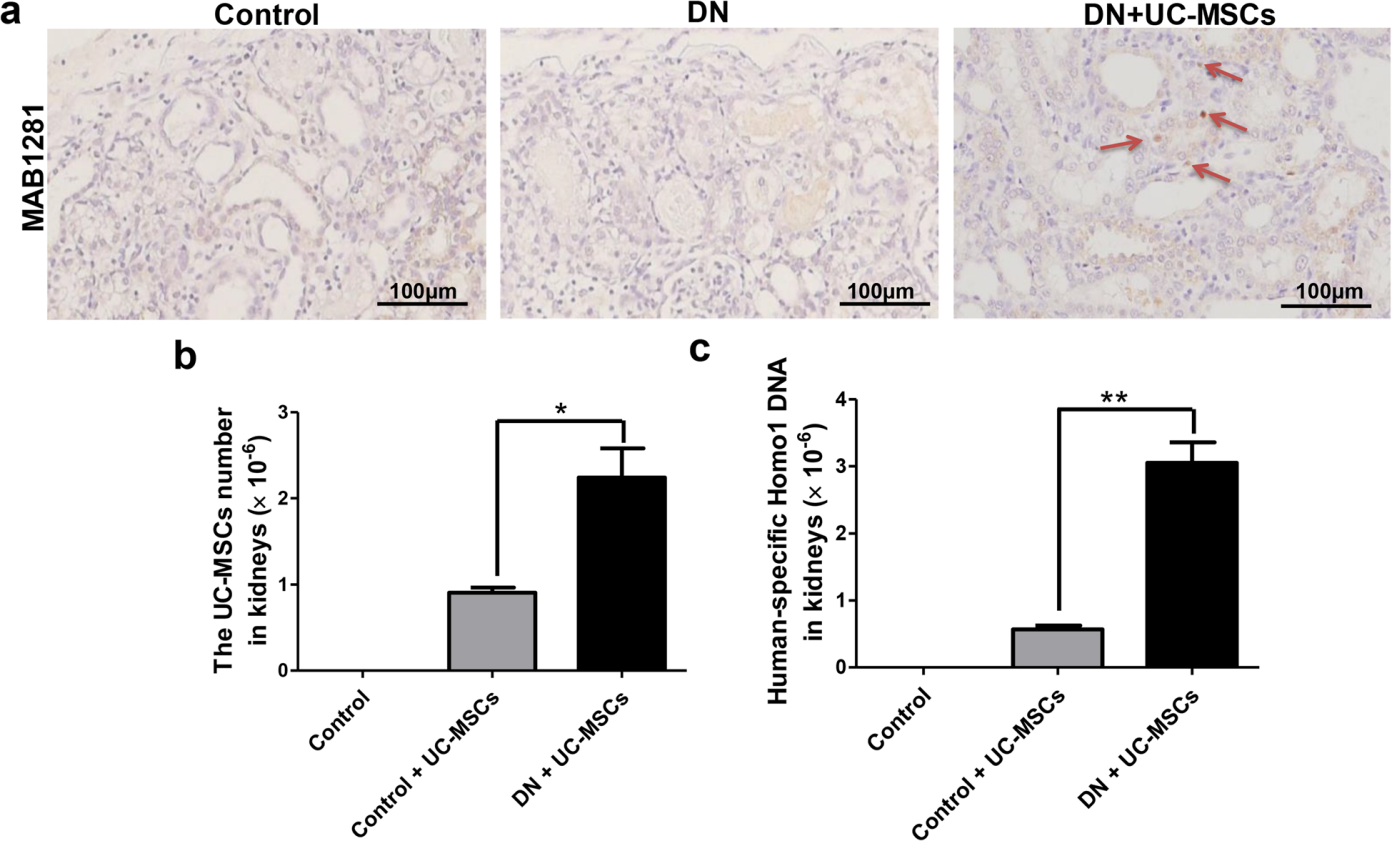
The location and distribution of UC-MSCs in kidneys of DN rats at 2 weeks after intravenous infusion. **a** The red arrow indicates MAB1281-positive cells in the kidneys of DN rats. **b** The number of UC-MSCs detected in the kidneys of DN rats. **c** Relative expression of human-specific Homol DNA in kidneys of DN rats. *N* = 3–4 (per group), data are presented as mean ± SEM. *P < 0.05, **P < 0.01 vs control +UC-MSC group

Although we did not detect significant differences in the body weight (Fig. 4a) and kidney weight (Fig. 4b), the kidney weight/kidney weight index was significantly decreased in the DN + UC-MSC group, compared with DN group (Fig. 4c). H&E staining showed that inflammatory cell infiltration occurred in the renal tubule interstitial tissue and granular degeneration in renal tubular epithelial cells in the DN group. PAS staining showed that the glomerular basement membrane was slightly thickened. Vacuolar degeneration of renal tubular epithelial cells appeared in both H&E and PAS staining of the DN. However, UC-MSC treatment alleviated the above pathological changes (Fig. 4d, e). To confirm whether UC-MSCs have migrated to the kidney tissues, immunohistochemistry staining of MAB1281 and RT-qPCR assay were performed as described [23], to detect the location and distribution of UC-MSCs in the kidneys of rats at 2 weeks after infusion. We observed a small number of MAB1281-positive cells with marked nuclei in brown in the DN + UC-MSC group. Interestingly, MAB1281-positive cells mainly localized in tubulointerstitium. No MAB1281-positive cell was found in the control group or DN group (Fig. 5a). The amount of UC-MSCs in the kidney tissues of DN rats was 0.0002%, which was higher than that in the control +UC-MSC group (Fig. 5b). The control group was assayed as a negative control. Meanwhile, the content of human-specific DNA fragment HOMO1 in the DN + UC-MSC group was 0.0003%, which was significantly higher than that in the control +UC-MSC group (Fig. 5c).

### UC-MSC treatment reduced inflammation in DN rats

**Fig. 6:**
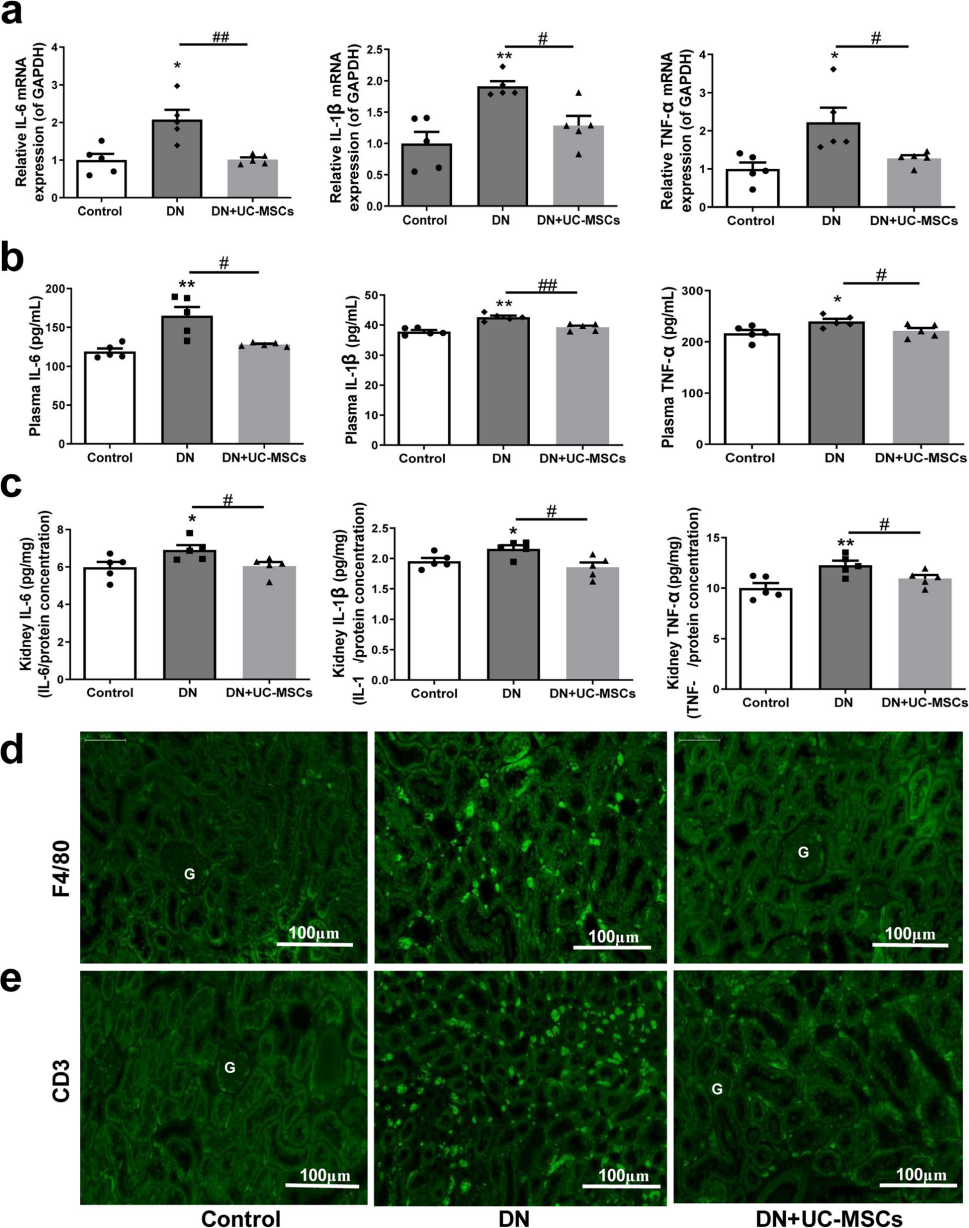
Effects of UC-MSCs on inflammatory response in DN rats. **a** Relative mRNA expression of IL-6, IL-1β, and TNF-α in kidney tissues. **b**, **c** Concentration of IL-6, IL-1β, and TNF-α in plasma and kidney tissues by ELISA. **d, e** Immunofluorescence staining of F4/80 (**d**) and CD3 (**e**) in kidney tissues. (G: glomerulus)*. N* = 5 (per group), data are presented as mean ± SEM. *P < 0.05, **P < 0.01 vs control. ^#^*P* < 0.05, ^##^*P* < 0.01 vs DN group

As shown in Fig. 6a–c, compared to the control group, the mRNA expressions of IL-6, IL-1β, and TNF-α (kidney tissues) and the concentrations of IL-6, IL-1β, and TNF-α (plasma and kidney tissues) were all significantly elevated in DN group, whereas these inflammatory cytokine levels were obviously reduced by UC-MSC treatment compared with DN group. F4/80 and CD3 immunofluorescence staining revealed that the infiltration of inflammatory cells mainly penetrated into the tubulointerstitium in the DN group, while UC-MSC treatment reduced the above pathological changes (Fig. 6d, e and Additional Fig. 1).

### UC-MSC treatment ameliorated renal fibrosis in DN rats

**Fig. 7:**
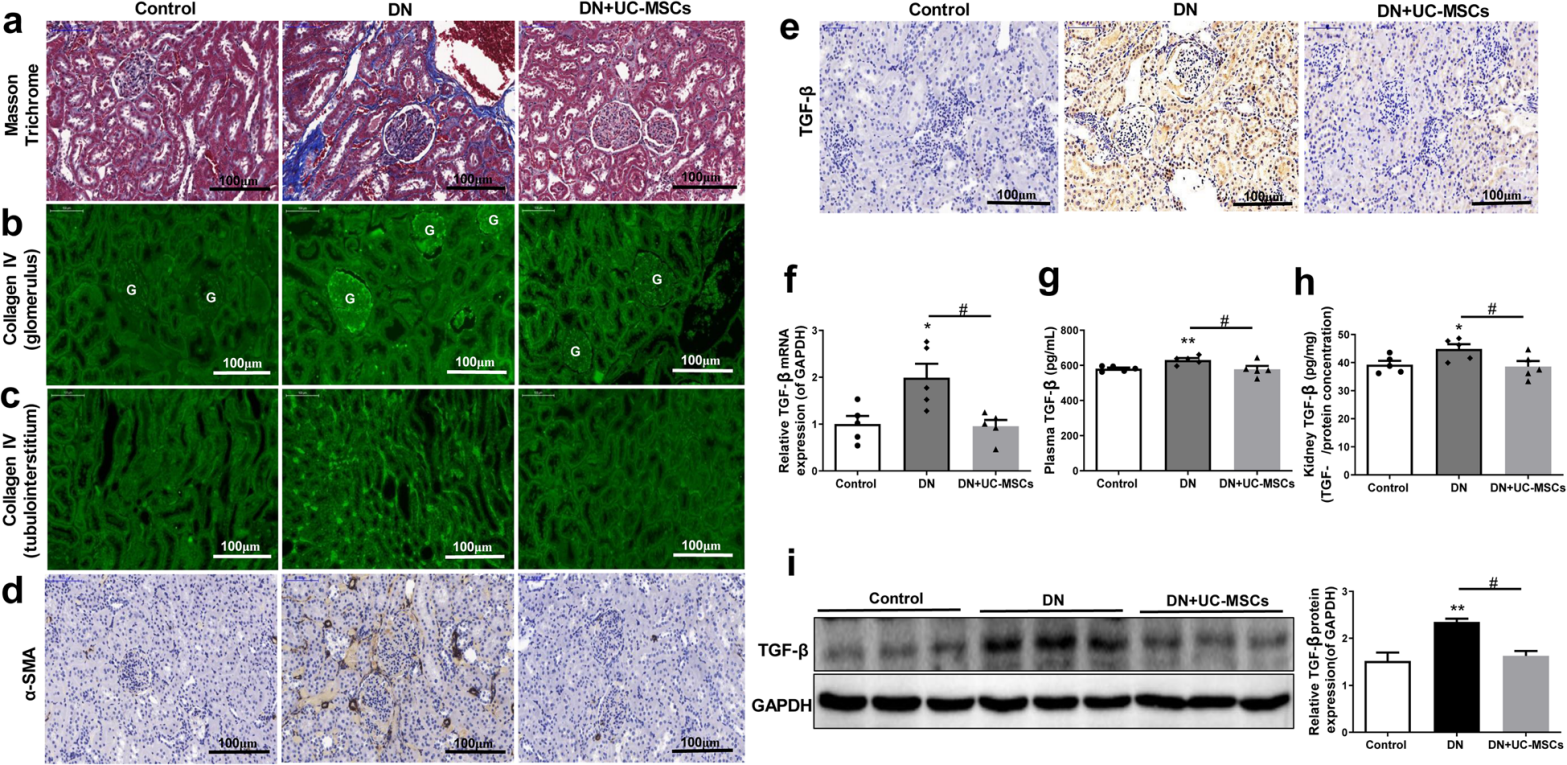
UC-MSC treatment alleviated glomerular and tubulointerstitial fibrosis in DN rats. **a** Masson’s trichrome staining. **b**, **c** Immunofluorescence staining of collagen IV in glomerulus (**b**) and tubulointerstitium (**e**). **d**, **e** Immunohistochemistry staining of α-SMA (**d**) and TGF-β (**e**) in kidney tissues. **f** The mRNA expression of TGF-β in kidney tissues. **g, h** Concentration of TGF-β in plasma (**g**) and kidney (**h**) tissues. **i** Western blot analysis of TGF-β in kidney tissues. *N* = 5 (per group), data are presented as mean ± SEM. *P < 0.05, **P < 0.01 vs control. ^#^*P* < 0.05 vs DN group

The Masson’s trichrome staining of renal tissues showed that renal glomerular and interstitial fibrosis occurred in the DN group, while the renal fibrosis was improved by UC-MSC treatment in DN + UC-MSC group (Fig. 7a). To further explore the possible role of UC-MSCs in anti-renal fibrosis, we examined the molecules involved in renal fibrosis. The immunofluorescence staining revealed that Collagen IV was majorly expressed in glomerular and tubulointerstitium in the DN group, while the expression of Collagen IV was greatly reduced in DN + UC-MSC group (Fig. 7b, c). Meanwhile, the immunohistochemistry staining showed that the expressions of α-SMA and TGF-β in the kidneys of DN rats were obviously increased; however, the expression of α-SMA and TGF-β were decreased after treatment with UC-MSCs (Fig. 7d, e). To further confirm that TGF-β was involved in renal fibrosis protection, RT-qPCR, ELISA, and Western blot assays were performed. We found that the mRNA (Fig. 7f) and protein (Fig. 7g–i) expression levels of TGF-β were markedly enhanced in the DN group, compared to the control group. However, UC-MSC treatment significantly inhibited this enhancement (Fig. 7f–i), further suggesting that the inhibition of TGF-β expression is an underlying mechanism of anti-renal fibrosis of UC-MSCs.

### UC-MSCs depressed cytokine expression in high glucose-injured renal tubular epithelial cells and renal glomerular endothelial cells

**Fig. 8:**
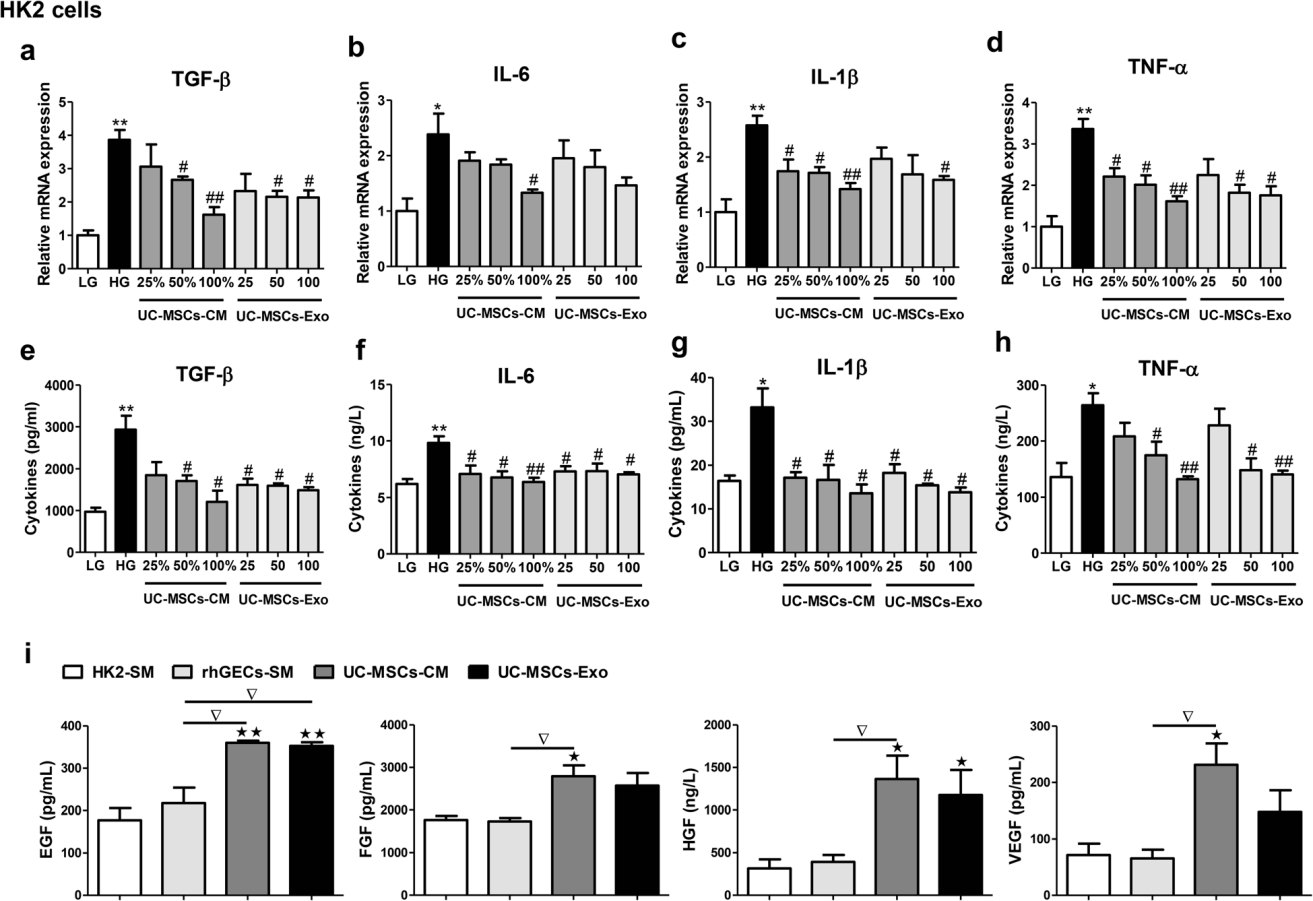
UC-MSC-CM or UC-MSC-Exo depressed cytokine expression in high glucose-injured HK2 cells. **a-d** mRNA expression of TGF-β (**a**), IL-6 (**b**), IL-1β (**c**) and TNF-α (**d**) in HK2 cells. **e-h** Concentration of TGF-β (**e**), IL-6 (**f**), IL-1β (**g**), and TNF-α (**h**) in the supernatant of HK2 cells. **i** Concentration of EGF, FGF, HGF, and VEGF in UC-MSC-CM and UC-MSC-Exo. *N* = 3 independent experiments. Data are presented as mean ± SEM. *P < 0.05, **P < 0.01 vs LG group; ^#^*P* < 0.05, ^##^*P* < 0.01 vs HG group; ^★^*P* < 0.05, ^★★^*P* < 0.01 vs HK2-SM group, ^▽^*P* < 0.05 vs hrGEC-SM group. UC-MSC-CM: UC-MSC conditioned medium; UC-MSC-Exo: UC-MSC-derived exosomes; LG: low glucose; HG: high glucose; SM: standard medium

To further evaluate the anti-inflammatory and anti-fibrotic effects of UC-MSCs, in vitro experiments were performed in HK2 cells (human proximal tubular epithelial cell line), NRK-52E cells (rat renal tubular epithelial cell line), and hrGECs (human renal glomerular endothelial cell line). UC-MSC-CM was collected, and UC-MSC-Exo was isolated and identified (Additional Fig. 2a–d). Compared with the controls, the mRNA expression and secretion of TGF-β, IL-6, IL-1β, and TNF-α were obviously upregulated in high glucose-treated HK2, NRK-52E, and hrGECs (Fig. 8a–h, Additional Figs. 3 and 4). However, UC-MSC-CM or UC-MSC-Exo significantly suppressed high glucose-induced production of TGF-β, IL-6, IL-1β, and TNF-α, in a dose-dependent manner (Fig. 8a–h, Additional Fig. 4).

To investigate whether UC-MSCs secreted anti-inflammation and anti-fibrosis-related factors, EGF, FGF, HGF, and VEGF levels were measured by ELISA. As shown in Fig. 8i, UC-MSCs secreted large amounts of EGF, FGF, HGF, and VEGF in both UC-MSC-CM and UC-MSC-Exo.

## Discussion

The current clinical routine treatment of DN mainly includes strict blood glucose control and hypertension control to prevent the development of DN. However, despite considerable educational efforts in controlling the disease, a large number of patients still develop not only early and mid-term DN, but also the end-stage chronic kidney disease. Currently, these therapies are still very limited and none of them can effectively cure DN [19]. In this study, we established a STZ-induced DN rat model and in vitro high glucose-induced HK2 cells, NRK-52E cells, and hrGEC injury models, then performed UC-MSC treatment to explore the efficacy and the underlying mechanisms of UC-MSCs in the treatment of DN. We confirmed that UC-MSCs could migrate to the kidneys and effectively repair renal dysfunction, including reducing the proteinuria, Scr, and urea nitrogen levels and elevating Ccr in DN rats. Importantly, UC-MSCs significantly reduce the inflammatory response and improve renal fibrosis of DN.

MSCs have many physiological functions, such as self-renewal, production of clonal cell populations, and multilineage differentiation [20]. In recent years, research on the treatment of DN with MSCs has focused on BM-MSCs [24–26]. In clinical applications, there are many problems with the source of BM-MSCs, including the severe pain of BM aspiration and the low number of collected cells, so this has led many researchers to look for other MSCs that are more readily available to replace BM-MSCs. UC-MSCs are a group of young adult MSCs derived from the neonatal umbilical cord tissue. It has the characteristics of the easy collection, greater proliferative capacity, and less antigenicity [20]. UC-MSCs have been used to study a variety of refractory diseases, such as diabetic foot [27], knee osteoarthritis [28], and premature ovarian failure [29]. UC-MSCs are becoming another promising MSCs for treating a variety of human refractory diseases.

Our results showed that, after UC-MSC transplantation, there was no significant change in the blood glucose in DN + UC-MSC group compared with the DN group. This result is consistent with the previous report [30], which may be due to the late treatment of MSCs and the missed opportunity for repairing the acute injury of the pancreas. The islets have been completely destroyed and could not be recovered. We found that 2 weeks after UC-MSC transplantation, compared with the DN group, the 24-h urinary protein concentration, Scr, urea nitrogen, and renal hypertrophy index of the DN + UC-MSC group were significantly reduced, while Ccr was significantly increased, suggesting that UC-MSCs had a protective effect on kidney function.

Histological changes in the kidneys are manifested by significant reductions in renal vacuole degeneration, inflammatory cell infiltration, and renal interstitial fibrosis after UC-MSC treatment. Meanwhile, after 2 weeks of intravenous infusion of UC-MSCs, we can detect a small number of cell colonization in rat kidney tissues, and the number of UC-MSCs in DN rats is more than that in normal rats. Previous studies have suggested that when the body ischemia, hypoxia, and injury, MSCs have a “homing” trait that predominately distributes to the injury site [31]. Many researchers believe that the homing ability of MSCs may be the key to their therapeutic effect. Some studies have reported that transplanted MSCs can be tracked in kidney tissues after MSC injection, suggesting that they may be involved in the repair of kidney damage through targeted differentiation or immune regulation [30, 32]. However, it should be noted that the number of “homing” of exogenous MSCs to the injury site is often limited and does not last long [33]. These results indicated that UC-MSCs can home to kidney tissues and improve the symptoms and pathological changes of DN to a certain extent.

The inflammatory cell infiltration and interstitial fibrosis in DN are associated with multiple inflammatory cytokines, such as IL-1β, IL-6, TNF-α, and TGF-β [21, 34, 35]. The expression of IL-1β, IL-6, and TNF-α was increased in the kidneys of DN rats. UC-MSC treatment significantly downregulate the mRNA expression of the above cytokines and reduce the secretion of these cytokines. In addition, UC-MSC treatment inhibited the recruitment of F4/80-labeled macrophages and CD3-labeled lymphocytes in kidney tissues and reduced the inflammatory response in DN rats. Macrophages are the main cellular source of TGF-β, which interfere with the cell cycle and cause kidney hypertrophy in early DN. TGF-β can regulate the gene transcription of myofibroblast phenotype, which is related to the transdifferentiation of renal tubular epithelial cells to fibroblasts and eventually lead to renal interstitial fibrosis [36, 37]. In this study, UC-MSCs obviously reduced the TGF-β level and downregulated the renal tubular expression of fibroblast markers, such as α-SMA and collagen IV in DN rats. Our results suggest that the improvement effect of UC-MSCs on renal fibrosis in DN rats might be closely associated with the TGF-β signaling pathway. The pathogenesis of DN is extremely complex and a variety of cytokines and growth factors are involved in its formation. Better understanding of the activation of TGF-β signaling pathway and its role in DN may provide novel tools for the prevention of renal fibrosis in the future.

To further elucidate the effect of UC-MSCs on DN, we performed in vitro experiments with high glucose-injured HK2 cells, NRK-52E cells, and hrGECs. As expected, we observed that high glucose stimulated the release of pro-inflammatory cytokines (IL-6, IL-1β, TNF-α) and pro-fibrotic factor (TGF-β) in HK2, NRK-52E, and hrGECs; however, UC-MSC-CM and UC-MSC-Exo significantly decreased high glucose-induced production of these cytokines in a dose-dependent manner. These results suggested that UC-MSCs suppressed inflammation and fibrosis via a paracrine mechanism. To further confirm this issue, we detected certain growth factors that are helpful for anti-inflammation and anti-fibrosis, including EGF, FGF, HGF, and VEGF. Our results indicated that UC-MSCs secreted large amounts of EGF, FGF, HGF, and VEGF in the UC-MSC-CM and UC-MSC-exo. Indeed, previous studies have reported that these growth factors exert anti-fibrotic and anti-inflammatory effects [38–40]. According to our observation in this study and other groups, we speculate that UC-MSC-derived EGF, FGE, HGF, and VEGF might participate in the anti-inflammatory and anti-fibrotic effect of UC-MSCs on DN. Further studies are required to shed light on the potential involvement and mechanism of these grow factor endocrine and paracrine profiles in DN. Our results indicate that reducing these inflammatory factors and fibrosis factor in DN kidneys can help improve renal function and greatly help understand the role of UC-MSCs in repairing renal function in DN.

## Conclusion

Our study demonstrated that UC-MSCs can effectively improve the renal function, inhibit inflammation and fibrosis, and prevent the progression of early DN. These findings provide a basis for the clinical use of UC-MSCs as a new therapeutic approach for DN. Further studies are needed to clarify the relationship between pharmacokinetics and efficacy/toxicity of UC-MSCs in DN and confirm the precise mechanisms of the anti-inflammatory and anti-fibrotic effects of UC-MSCs in improving the occurrence and development of DN.

## Supplementary information

**Additional file 1: Table S1.** Primers sequences and RT-qPCR conditions for rat genes. **Table S2.** Primers sequences and RT-qPCR conditions for human genes.

**Additional file 2: Figure S1.** Immunofluorescence staining of F4/80 and CD3 in the glomerulus of DN kidney tissues. G: glomerulus. **Figure S2.** Isolation and identification of exosomes extracted from UC-MSCs (UC-MSCs-Exo). **Figure S3.** UC-MSCs-CM depressed cytokine expression in high glucose-injured NRK-52E cells. **Figure S4.** UC-MSCs-CM or UC-MSCs-Exo depressed cytokine expression in high glucose-injured hrGECs.

## Data Availability

All data generated and/or analyzed during this study are included in this published article.

## Abbreviations

DN: Diabetic nephropathy
MSCs: Mesenchymal stem cells
UC-MSCs: Human umbilical cord MSCs
BM-MSCs: Bone marrow MSCs
AD-MSCs: Adipose-derived MSCs
STZ: Streptozotocin
UC-MSC-CM: UC-MSC-conditioned medium
UC-MSC-Exo: UC-MSC exosomes
HG: High glucose
LG: Low glucose
IL-6: Interleukin 6
IL-1β: Interleukin 1β
TNF-α: Tumor necrosis factor-α
TGF-β: Transforming growth factor β
α-SMA: α-Smooth muscle actin
MAB1281: Mouse anti-human nuclei monoclonal
HOMO1: Human-specific DNA fragment
NRK-52E: Renal tubular epithelial cells
HK2 cells: Human proximal tubular epithelial cells
hrGECs: Human renal glomerular endothelial cells
EGF: Epidermal growth factor
FGF: Fibroblast growth factor
HGF: Hepatocyte growth factor
VEGF: Vascular endothelial growth factor
SM: Standard medium
