# Supplementary material for: Human umbilical cord-derived mesenchymal stem cells prevent the progression of early diabetic nephropathy through inhibiting inflammation and fibrosis
Source: Stem Cell Res Ther. 2020 Aug 3;11:336. doi: 10.1186/s13287-020-01852-y (PMC7397631; doi:10.1186/s13287-020-01852-y)
Supplement: Supplementary file 1 — Additional file 1: Table S1. Primers sequences and RT-qPCR conditions for rat genes. Table S2. Primers sequences and RT-qPCR conditions for human genes. [file 13287_2020_1852_MOESM1_ESM.docx]

**Additional Tables**

**Human umbilical cord-derived mesenchymal stem cells prevent the progression of early diabetic nephropathy through inhibiting inflammation and fibrosis**

E Xiang^1, 2^, Bing Han ^2^, Quan Zhang^2^, Wei Rao^2^, Zhangfan Wang^2^, Cheng Chang^1^, Yaqi Zhang^1^, Chengshu Tu^2^, Changyong Li^3*^, and Dongcheng Wu ^1, 2*^

1Department of Biochemistry and Molecular Biology, Wuhan University School of Basic Medical Sciences, Wuhan, China

2Wuhan Hamilton Biotechnology Co., Ltd, Wuhan, China

3Department of Physiology, Wuhan University School of Basic Medical Sciences, Wuhan, China

* Correspondence should be addressed to Dongcheng Wu ([bcdcwu@hotmail.com](mailto:bcdcwu@hotmail.com)) and Changyong Li (lichangyong@whu.edu.cn).

**Additional table 1** Primers sequences and RT-qPCR conditions for rat genes

| Genes | Forward primer | Reverse primer |
| --- | --- | --- |
| TGF-β1 | TCTAAGGATGAGGAGTGGAAGA | CTCCATACTGTTCGATGACAGG |
| IL-1β | GTCAGTAGTGACGATTCCAACA | TTCCCTTGTTCTCCTTGCTAAA |
| IL-6 | CTGCATTGGCATGAGGTTTG | TCAGAGGGATCTGTGTCTTCT |
| TNF-α | GAGCCACGGGCTATCATTTC | CTCCCAGCAAACGGACAGAT |
| GAPDH | GCAAGTTCAACGGCACAG | GCCAGTAGACTCCACGACA |

TGF-β: transforming growth factor β; IL-1β: interleukin 1β; IL-6: interleukin 6; TNF-α: tumor necrosis factor-α; GAPDH, glyceraldehyde phosphate dehydrogenase.

**Additional table 2** Primers sequences and RT-qPCR conditions for human genes

| Genes | Forward primer | Reverse primer |
| --- | --- | --- |
| TGF-β1 | TTGATGTCACCGGAGTTGTG | GTAGTGAACCCGTTGATGTCC |
| IL-1β | TGGCCCTAAACAGATGAAGTG | ATCTTCCTCAGCTTGTCCATG |
| IL-6 | CCACTCACCTCTTCAGAACG | CATCTTTGGAAGGTTCAGGTTG |
| TNF-α | ATGAGCACTGAAAGCATGATCC | GAGGGCTGATTAGAGAGAGGTC |
| GAPDH | GAAATCCCATCACCATCTTCCAG | ATGAGTCCTTCCACGATACCAAAG |

TGF-β: transforming growth factor β; IL-1β: interleukin 1β; IL-6: interleukin 6; TNF-α: tumor necrosis factor-α; GAPDH, glyceraldehyde phosphate dehydrogenase.
