# Supplementary material for: Human umbilical cord-derived mesenchymal stem cells prevent the progression of early diabetic nephropathy through inhibiting inflammation and fibrosis
Source: Stem Cell Res Ther. 2020 Aug 3;11:336. doi: 10.1186/s13287-020-01852-y (PMC7397631; doi:10.1186/s13287-020-01852-y)
Supplement: Supplementary file 2 — Additional file 2: Figure S1. Immunofluorescence staining of F4/80 and CD3 in the glomerulus of DN kidney tissues. G: glomerulus. Figure S2. Isolation and identification of exosomes extracted from UC-MSCs (UC-MSCs-Exo). Figure S3. UC-MSCs-CM depressed cytokine expression in high glucose-injured NRK-52E cells. Figure S4. UC-MSCs-CM or UC-MSCs-Exo depressed cytokine expression in high glucose-injured hrGECs. [file 13287_2020_1852_MOESM2_ESM.zip › Additional file 2-Figures.docx]

**Additional Figures**

**Human umbilical cord-derived mesenchymal stem cells prevent the progression of early diabetic nephropathy through inhibiting inflammation and fibrosis**

E Xiang^1, 2^, Bing Han ^2^, Quan Zhang^2^, Wei Rao^2^, Zhangfan Wang^2^, Cheng Chang^1^, Yaqi Zhang^1^, Chengshu Tu^2^, Changyong Li^3*^, and Dongcheng Wu ^1, 2*^

1Department of Biochemistry and Molecular Biology, Wuhan University School of Basic Medical Sciences, Wuhan, China

2Wuhan Hamilton Biotechnology Co., Ltd, Wuhan, China

3Department of Physiology, Wuhan University School of Basic Medical Sciences, Wuhan, China

* Correspondence should be addressed to Dongcheng Wu ([bcdcwu@hotmail.com](mailto:bcdcwu@hotmail.com)) and Changyong Li (lichangyong@whu.edu.cn).

**Additional figure captions:**

**Additional figure 1** Immunofluorescence staining of F4/80 and CD3 in glomerulus of DN kidney tissues. G: glomerulus.

**Additional figure 2** Isolation and identification of exosomes extracted from UC-MSCs (UC-MSCs-Exo). **a** Flowchart of UC-MSCs-Exo extraction. **b** UC-MSCs-Exo was observed by transmission electron microscope (TEM). Magnification: ×11500. **c** Representative marker CD9, Alix of UC-MSCs-Exo. Calnexin was used as negative control. Both lanes were loaded with 50 μg proteins as measured. **d** Nanoparticle tracking analysis (NTA) of size distribution and concentration of UC-MSCs-Exo. *N*=3 independent experiments.

**Additional figure 3** UC-MSCs-CM depressed cytokine expression in high glucose-injured NRK-52E cells. a-d mRNA expression of TGF-β, IL-6, IL-1β and TNF-α in NRK-52E cells. e-h Concentration of TGF-β, IL-6, IL-1β and TNF-α in the supernatant of NRK-52E cells. *N*=3 independent experiments. Data are presented as mean ± SEM. **P* < 0.05, ***P* < 0.01 *vs* LG group, ^#^*P* < 0.05 *vs* HG group. LG: low glucose; HG: high glucose.

**Additional figure 4** UC-MSCs-CM or UC-MSCs-Exo depressed cytokine expression in high glucose-injured hrGECs. **a**-**d** mRNA expression of TGF-β (**a**), IL-6 (**b**), IL-1β (**c**)and TNF-α(**d**) in hrGECs. **e**-**h** Concentration of TGF-β (**e**), IL-6 (**f**), IL-1β (**g**) and TNF-α (**h**) in the supernatant of hrGECs. *N*=3 independent experiments. Data are presented as mean ± SEM. **P* < 0.05, ***P* < 0.01 *vs* LG group, ^#^*P* < 0.05, ^##^*P* < 0.01 *vs* HG group. LG: low glucose; HG: high glucose.
